# Treatment of granulomatous perioral dermatitis with 1.5% topical ruxolitinib cream

**DOI:** 10.1016/j.jdcr.2024.02.022

**Published:** 2024-03-06

**Authors:** Sophia S. Tran, Benjamin Ungar, Patrick M. Brunner

**Affiliations:** Department of Dermatology, Icahn School of Medicine at Mount Sinai, New York, New York

**Keywords:** eczema, perioral dermatitis, ruxolitinib, skin side effect, steroid side effect

## Introduction

Perioral dermatitis (POD) is an inflammatory skin condition characterized by facial eruptions of small papules, papulovesicles, and/or pustules with erythema and scaling, typically around the perioral area, but can also involve perinasal and periorbital areas (“periorifical dermatitis”).^1^ POD may also manifest as its clinical variant known as granulomatous POD, characterized by small, flesh-colored to red-brown inflammatory papules.^2^ Generally, POD has commonly been reported in young adult females and children but the exact annual incidence is unknown and can vary with geographic location.^3^

Long-term topical corticosteroid use is a typical elicitor of POD.^4^ This eruption is initially responsive to topical steroid treatment, but typically exacerbates upon withdrawal of the treatment.^5^ Underlying pathomechanisms by which topical corticosteroid treatment predisposes patients to POD are unclear, but it is assumed that they disrupt the skin barrier by influencing the microflora of the hair follicle or triggering infections including *Candida albicans*, fusiform bacteria, and *Demodex* mites.^1^

Several treatment modalities for POD have been developed based on expert consensus. Importantly, topical corticosteroids need to be discontinued, and alternative topical treatments, such as antibiotics and calcineurin inhibitors, are usually prescribed.^6^ In more severe cases, systemic treatments such as oral tetracyclines are given.^7^ However, a considerable number of patients respond insufficiently to these agents or experience adverse events.^6^ Oral tetracyclines, in particular, may be contraindicated and raise concerns, especially in pediatric patients under 8 years of age for increased risk of permanent teeth discoloration, impaired bone growth, and photosensitivity.^1^ Thus, there is a high need for the development of better and safer therapies.

Ruxolitinib, a Janus kinase (JAK) inhibitor, has recently been proven effective for the treatment of several inflammatory diseases including atopic dermatitis (AD) and vitiligo,^8^^,^^9^ but there are currently no published reports on the use of topical ruxolitinib for POD. We present a case that highlights off-label use of 1.5% topical ruxolitinib cream as a possible treatment modality for patients with POD.

## Case report

An 18-year-old woman presented with a history of a rash on her face for several months, located primarily around the mouth and lower eyelids. The patient reported that this rash was nonresponsive to triamcinolone 0.1% ointment, which she had been using for underlying AD for 5 months. Besides AD, the patient had a medical history that included allergic asthma, childhood eczema as well as food allergies to sesame and tree nuts that previously resulted in anaphylaxis.

**Fig 1:**
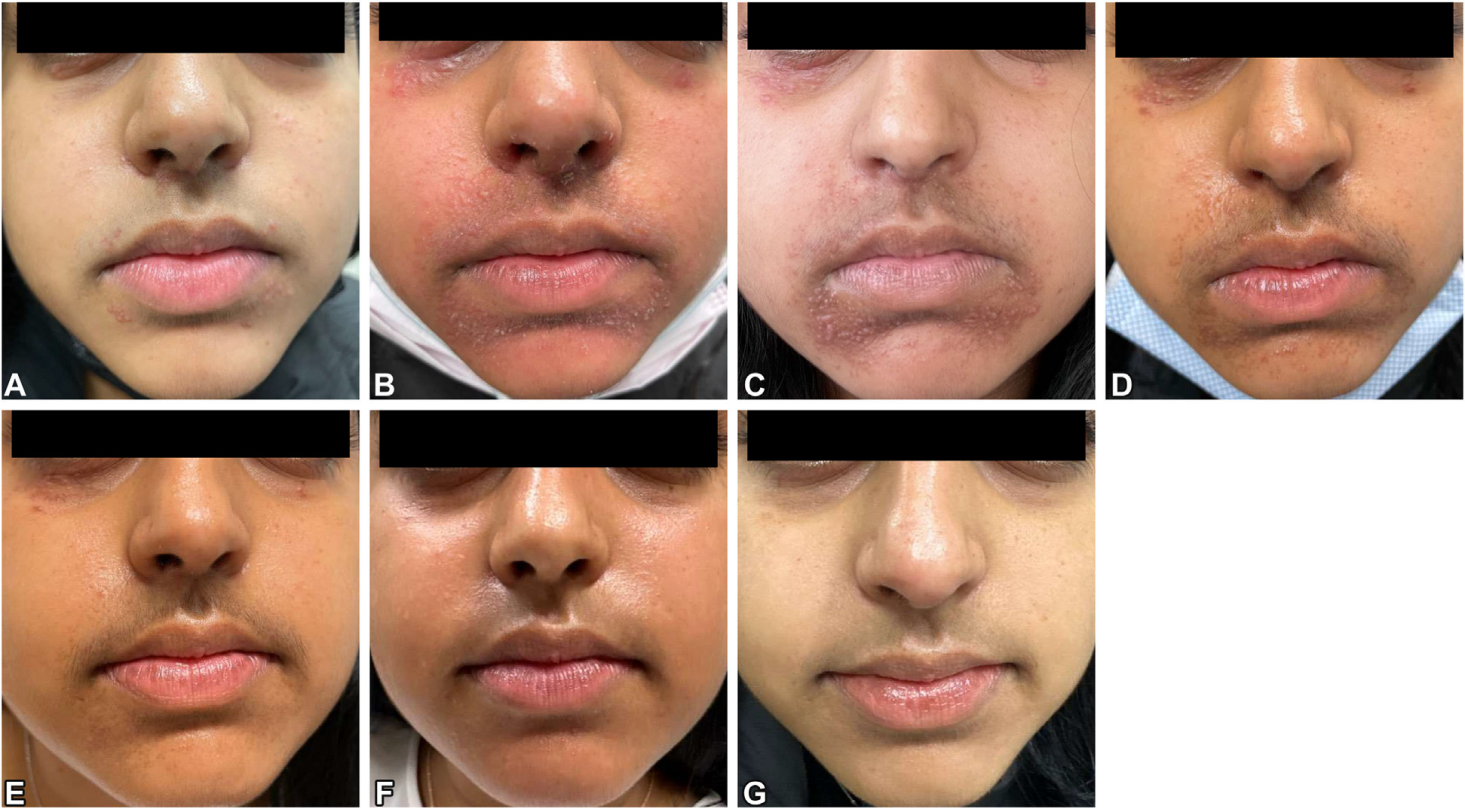
An 18-year-old woman with steroid-induced perioral dermatitis (POD) located on the perioral, perinasal, and lower eyelid areas. **A,** Initial presentation; ongoing treatment with triamcinolone 0.1% ointment for 5 months. **B,** POD flare after 4 weeks of topical steroid withdrawal, with *Staphylococcus aureus* superinfection. **C,** Crusting improved after a 10-day course of amoxicillin/clavulanate 875/125 mg, topical pimecrolimus 1% cream, and topical mupirocin 2% ointment, but with unchanged granulomatous POD. **D-G,** Clinical appearance after (**D**) 1 week, (**E**) 7 weeks, (**F**) 20 weeks, and (**G**) 34 weeks of topical 1.5% ruxolitinib treatment, with complete resolution of POD and normalization of postinflammatory hyperpigmentation.

On physical examination, the patient was noted to have a perioral rash consisting of several small, flesh-colored to red-brown inflammatory papules in a perioral, perinasal, and periocular fashion (Fig 1, *A*). Given the patient’s previous long-term use of triamcinolone cream on her face, the findings were clinically consistent with granulomatous POD. The patient was initially advised to stop steroid use and to limit skin treatment to topical moisturizers, which led to disease exacerbation within the following 4 weeks, accompanied by considerable crusting (Fig 1, *B*). A skin swab was performed, and the patient was started on topical pimecrolimus 1% cream twice a day for exacerbation of POD, and mupirocin 2% ointment twice a day for suspected bacterial superinfection. Bacterial culture results confirmed the presence of *Staphylococcus aureus* that was resistant to tetracyclines, erythromycin, and clindamycin, prompting us to prescribe amoxicillin/clavulanate 875/125 mg twice a day for 10 days.

After completing this 10-day course of antibiotics combined with topical pimecrolimus 1% cream, the patient noted resolution of crusting, but otherwise very little improvement in the affected areas (Fig 1, *C*). Given resistance to conventional treatment approaches, the patient was prescribed off-label use of topical ruxolitinib 1.5% cream twice a day. The patient tolerated the medication very well and showed prompt improvement after 1 week of therapy (Fig 1, *D*), with further resolution over the next 7 weeks, resulting in very few residual papules and some postinflammatory hyperpigmentation (Fig 1, *E*). Continued use of topical ruxolitinib led to further improvement, as evidenced after 20 weeks (Fig 1, *F*). After 34 weeks of topical ruxolitinib use, the patient noticed that her POD had completely resolved, with normalization of skin pigmentation (Fig 1, *G*), and treatment was stopped. So far, the patient has not reported any relapse of her condition.

## Discussion

Ruxolitinib, a JAK inhibitor, works by specifically blocking JAK1 and JAK2 protein kinases, thereby inhibiting the downstream signal transducer and activator of transcription pathway to reduce inflammation in the skin.^8^ In the reported case, topical ruxolitinib was highly effective in treating POD, with marked improvement within 7 weeks and complete resolution after 34 weeks of continuous application. We hypothesize that in this patient, long-term topical corticosteroid use had contributed to critical upregulation of the JAK/signal transducer and activator of transcription pathway, which was essential for the formation of her POD. As such, future studies investigating JAK inhibitors should be considered to help establish a new line of treatment for patients with this often prolonged and debilitating condition.

Local adverse events associated with topical ruxolitinib are usually mild to moderate and include application-site acne and pruritus.^8^, ^9^, ^10^ However, no adverse events were reported by the patient while using topical ruxolitinib. However, there is a lack of sufficient data to assess its long-term safety and its potential long-term effects on POD. It is important to note that topical ruxolitinib has only been recently approved for treating AD and vitiligo in patients aged ≥12 years.^8^^,^^10^ The medication has been approved for the short-term and noncontinuous management of AD in nonimmunocompromised patients^8^ as well as for the treatment of nonsegmental vitiligo.^10^ Given these considerations, topical ruxolitinib use for POD appears promising, however, additional research will be necessary to evaluate its safety and efficacy in this context.

From this case report, we conclude that topical ruxolitinib 1.5% cream should be studied in future clinical trials as an alternative treatment option for patients with POD who are unresponsive to primary therapies and/or have contraindications to established treatments.

## Conflicts of interest

None disclosed.
